# Hate Speech Against Asian American Youth: Pre-Pandemic Trends and The Role of School Factors

**DOI:** 10.1007/s10964-024-01987-8

**Published:** 2024-05-04

**Authors:** Kevin A. Gee, North Cooc, Peter Yu

**Affiliations:** 1 School of Education, University of California, Davis, USA; 2 College of Education, University of Texas, Austin, USA

**Keywords:** Hate speech, Asian American Adolescents, Authoritative school climate, National Crime Victimization Survey, School Crime Supplement, Logistic regression.

## Abstract

Although hate speech against Asian American youth has intensified in recent years—fueled, in part, by anti-Asian rhetoric associated with the COVID-19 pandemic—the phenomenon remains largely understudied at scale and in relation to the role of schools prior to the pandemic. This study describes the prevalence of hate speech against Asian American adolescents in the US between 2015 and 2019 and investigates how school-related factors are associated with whether Asian American youth are victims of hate speech at school. Analyses are based on a sample of 938 Asian American adolescents (*M*_age_ = 14.8; 48% female) from the three most recently available waves (2015, 2017, and 2019) of the School Crime Supplement to the National Crime Victimization Survey. On average, approximately 7% of Asian Americans were targets of hate speech at school between 2015 and 2019, with rates remaining stable over time. Findings also indicate that students had lower odds of experiencing hate speech if they attended schools with a stronger authoritative school climate, which is characterized by strict, yet fair disciplinary rules coupled with high levels of support from adults. On the other hand, Asian American youth faced higher odds of experiencing hate speech if they were involved in school fights. Authoritative school climate and exposure to fights are malleable and can be shaped directly by broader school climate related policies, programs and interventions. Accordingly, efforts to promote stronger authoritative climates and reduce exposure to physical fights hold considerable potential in protecting Asian American youth from hate speech at school.

## Introduction

The COVID-19 pandemic unleashed not only a widespread and unprecedented global health crisis, but social divisiveness ensued, intensifying and amplifying hate particularly against Asians and Asian Americans who were unjustly blamed and scapegoated for the pandemic (Lim et al., 2023). The reported surge in anti-Asian American and Pacific Islander (AAPI) hate involved a range of discriminatory acts, including hate speech, a form of expression that perpetrators deliberately use to demean, devalue, and exclude individuals and groups based on their identities (e.g., race, ethnicity, disability status, gender, etc.; Wachs et al., 2022). Hate speech experienced in the broader AAPI community in the wake of the pandemic knew no boundaries and figured prominently in the lives of children and adolescents—about three out of every four 12–18 year olds who reported anti-Asian discrimination to Stop AAPI Hate experienced verbal harassment or name calling related to their race (Jeung et al., 2021). Although hate directed at Asian Americans has existed for generations (Lee, 2015), a prominent gap in the extant literature base is how pervasive hate speech was in the years *prior* to the pandemic among Asian American adolescents and ways in which schooling contexts could perpetuate it or offer protection from it. To fill this void in the current evidence base, this study describes the prevalence of hate speech among Asian American adolescents in the US leading up to the pandemic (2015–2019) and investigates how school contexts related to whether Asian American youth experienced hate speech at school.

### Hate Speech Among Adolescents: Theory and Evidence

Though there is no singular agreed upon definition of hate speech, it is widely understood to involve derogatory remarks that intentionally demean and harm individuals or groups of individuals based on characteristics such as their race, ethnicity, sexual orientation, gender, etc. (Kansok-Dusche et al., 2023). Perpetrators of hate speech intentionally attack others via words, images, or videos. While hate speech can be directed at others in-person, it has increasingly tended to proliferate through online platforms (Dowd et al., 2006). It is important to note that there are overlaps between definitions of bullying and hate speech in the theoretical and empirical literature as they both involve the intent to harm and devalue others (Kansok-Dusche et al., 2023). However, in contrast to incidents of hate speech, bullying can also be physical in nature and can be tied to factors besides race, ethnicity, sexual orientation, etc.; importantly, bullying occurs in the context of a social relationship where the victim is less powerful than the victimizer (i.e., asymmetric power imbalance) and incidents are typically repeated over time (American Psychological Association, 2024).

The pervasiveness of hate speech, be it in-person or online, is concerning given its negative consequences on adolescents’ outcomes. Developmentally, there is a strong positive relationship between online hate speech victimization and depressive symptoms (Wachs et al., 2022). In terms of school-related outcomes, increased exposure to verbal hate speech and observed hate speech, such as hateful graffiti, is associated with avoiding school as a whole and avoiding specific locations in school like the entrance and cafeteria (Lehman, 2020). School avoidance puts adolescents at a higher risk for decreased academic performance and educational attainment (Balfanz & Byrnes, 2012). Finally, youth who have been exposed to hate speech show less sensitivity to it, thereby leading to higher levels of outgroup prejudice and anti-immigrant attitudes (Soral et al., 2018).

Conceptually, insights from social learning theory alongside social norms theory helps shed light on why in-person hate speech can occur among adolescents, especially within schools (Wachs et al., 2022). Hate speech is conceptualized as a learned behavior (Wachs et al., 2022) that manifests through observing hate speech in one’s own social environments (e.g., schools). While not every adolescent who witnesses hate speech will go on to be perpetrators of it, two underlying conditions contribute to hate speech (Wachs et al., 2022). First, adolescents individually need to have the motivation to imitate and enact it. Underlying motivators, such as revenge, ideology, and group conformity, are common reasons for perpetrating hate speech (Wachs et al., 2022). Second, sets of social norms dictating how hate speech is tolerated in adolescents’ social environments need to be in place for hate speech to occur. Hate speech is more likely to occur if it is deemed acceptable in the social environment (i.e., injunctive hate-speech norms) and there is strong peer pressure to engage in it. Adolescents who believed that hate speech was an unacceptable behavior in the classroom were less likely to be perpetrators of hate speech, while deviant peer pressure (i.e., classmates urging the student to perform an unacceptable behavior) was positively associated with hate speech perpetration (Wachs et al., 2022). Further, the positive association between witnessing hate speech and perpetration was moderated by the injunctive hate-speech norms and deviant peer pressure. For students reporting that hate speech was less acceptable in the classroom, witnessing hate speech was associated with lower levels of perpetration. On the other hand, students who reported higher levels of deviant peer pressure were more likely to engage in hate speech if they witnessed it. To summarize, hate speech perpetration in schools is influenced both by a student’s individual motivators as well as the hate speech norms in the broader school context.

Beyond the theoretical underpinnings of hate-speech in schools, the actual encounters of hate speech that adolescents confront in their daily lives can take many forms, both online and in person, including witnessing hateful symbols or graffiti and witnessing offensive statements (Van Dorn, 2004). Further, where incidents of hate speech take place in schools can vary, with classrooms and break areas being the most common locations (Castellanos et al., 2023). Finally, a recent study examining hate speech among adolescents in Germany and Switzerland found that students’ skin color and country of origin were the most common targets of hate speech, both online and in-person (Castellanos et al., 2023).

### Hate Speech Against Asian American Youth

Though the US lacks a formal nationwide tracking system documenting incidents of hate speech against Asian Americans, recent data collection and reporting efforts by Stop AAPI Hate offer insights into the pervasiveness of hate speech in the lives of Asian Americans. Between March 2020 and March 2022, 63% of reported incidents (*n* = 11,467) involved verbal hate speech and/or harassment, the highest of all forms of AAPI hate (other forms include physical assault comprising 17% of reported incidents and avoidance or shunning at 16%; Stop AAPI Hate, 2022). Among a smaller sample of approximately 340 Asian American 12–18 year olds who reported incidents between March and July 2020, about three out of four were targets of verbal harassment or name-calling related to their race (Jeung et al., 2021). While racial identity comprises only one dimension of the Asian American experience, race appears to be one of the most salient characteristics of hate speech incidents, and is deeply rooted in xenophobia which has been further exacerbated by broader social and political rifts that have scapegoated AAPIs for a range of societal problems, from the past economic downturns (Lee, 2015) to the present day pandemic (Findling et al., 2022). Present day anti-AAPI hate speech is a manifestation and continuation of longer-term historical trends in racial discrimination against AAPI communities in the US that includes early anti-Asian immigrant initiatives, policies, and practices in the mid-nineteenth century which forcibly excluded Asians from political, economic, and social spheres of mainstream US society (Lee, 2015). During this period, Asians in America were perceived as anathema to Western civilization and ideals and were derogatorily referred to as the “Yellow Peril”, a racist phrase that was not only used to marginalize Asians from a rhetorical standpoint, but had real-world implications for their treatment and livelihoods (Lee, 2015).

### Schools and Hate Speech: Conceptual Framework

While current theory and evidence on hate speech among adolescents has elucidated how it is a learned behavior that can be further perpetuated by broader injunctive norms and deviant peer pressure, only recently has a deeper understanding of the role of schools in either perpetuating or protecting teens from hate speech been developed. Classroom climate, especially when characterized by stronger social cohesion and more positive social interactions, can serve as a protective factor against acts of hate speech because students are more likely to speak out against such hate (Wachs et al., 2023). At the school level, positive student-teacher relationships and stronger disciplinary structure and rule fairness were found to be negatively associated with hate speech (Lehman, 2019). In contrast, facets of formal social control, such as frequent punishment by school officials and presence of security guards in schools, were positively associated with hate speech. Importantly, these findings were correlational, so causation and directionality cannot be inferred; it is entirely possible that some schools increased formal social control in response to higher levels of hate speech.

In light of this evidence linking schools to hate speech, this study conceptualizes the link between school contexts and incidents of hate speech perpetrated against Asian American youth by drawing upon two interrelated frameworks that were originally conceived of to examine the link between schools and bullying victimization: Authoritative Disciplinary Theory (Gregory et al., 2010); and Opportunity Theory (Popp, 2012). Authoritative Disciplinary Theory posits that schools with caring and supportive adults (e.g., teachers), coupled with strong structure, in the form of clear and firm school rules, can lead to lower levels of victimization. Relatedly, Opportunity Theory suggests that victimization is influenced by the presence of guardianship (e.g., caring peers or adults), a victim’s exposure and proximity to victimizers (e.g., the presence of gangs at school), and a victimizer’s perception of their victim’s vulnerability (e.g., participation in certain school extracurricular activities that can suggest higher or lower levels of vulnerability to others).

In the context of Asian American victimization, prior evidence (Gee & Cooc, 2019) confirms these underlying theories—stronger guardianship, in the form of peer support, is linked to lower levels of physical victimization while exposure to gangs and physical fights is associated with increased incidents of social victimization. These findings complement those from the literature on school victimization of Asian American youth demonstrating that opportunity theory, in the form of participation in certain extracurriculars (e.g., athletics) can lead to higher levels of victimization (Peguero et al., 2015; Peguero & Williams, 2013). However, studies have yet to examine how either of these theories operates in the context of hate speech incidents against Asian American youth.

## Current Study

Although hate speech against Asian American youth is a growing phenomenon that has intensified in recent years—fueled, in part, by anti-Asian rhetoric associated with the pandemic—current attention to this phenomenon has overlooked what occurred before the pandemic. Knowledge of prior trends establishes a baseline with which to contextualize present and future trends. Further, research on how schools are linked to hate speech incidents is scarce. Schools can serve as sites of both perpetration and protection and importantly, given the large-scale reach into the daily lives of adolescents, pinpointing protective factors can yield insights into critical school features that are malleable and amenable to shifts in policies or practices that could ultimately benefit the wellbeing of Asian American youth. Accordingly, this study aims to answer two critical, yet unanswered questions about hate speech and Asian American adolescents in the US between 2015 and 2019. What was the prevalence of hate speech incidents between 2015 and 2019 and did the prevalence change over time? (Research Question 1). How do schools—particularly, their authoritative climates (i.e., supportive adults coupled with strong school rules) alongside factors related to victimization exposure (e.g., presence of gangs)—relate to the incidence of hate speech? (Research Question 2). Based on prior empirical and theoretical evidence linking authoritative climates to reductions in bullying victimization among Asian American youth, this study hypothesizes that the protective benefits of authoritative climate will extend to incidents of hate speech as well.

## Methods

### Dataset and Sample

Data for this study comes from the three most recently available waves (2015, 2017, and 2019) of the School Crime Supplement (SCS) to the National Crime Victimization Survey (NCVS), a large-scale nationwide survey that captures school victimization experiences of adolescents aged 12–18 (United States Bureau of Justice Statistics, 2021). The SCS was jointly developed by the National Center for Education Statistics (NCES) and the Bureau of Justice Statistics. The NCVS consists of a nationally representative sample of approximately 150,000 households and within those households, the SCS is administered as a supplemental survey to 12–18 year olds who are enrolled in either public or private schools. The SCS is administered every two years to approximately 6500 adolescents whose households were sampled to participate in the NCVS and asks adolescents about their experiences and perceptions of crime and safety at school, including their exposure to incidents of hate speech. The NCVS uses a series of incoming and outgoing rotation groups for sample households. Households in the incoming group remain in the sample for 3 years after which they exit and are replaced by new sample households. The SCS data includes responses from the incoming rotation group of households and thus represent distinct samples of adolescents in each wave (DeVoe & Bauer, 2011). Respondents eligible to be included in the SCS sample include those aged 12–18 who are currently enrolled in primary or secondary schools and their responses are collected via computer-assisted personal interviewing (Thomsen et al., 2024). Data were pooled together from the three most recent waves of the SCS, yielding an overall sample of *N* = 18,673 adolescents. From this pooled dataset, the analytic subsample consists of 938 adolescents who self-identified as Asian American (*n* = 244 in 2015; *n* = 340 in 2017; and *n* = 354 in 2019). Given that the data are anonymous and publicly available, this study was considered non-human subject research and not subject to Institutional Review Board review.

### Measures

#### Hate speech

The primary outcome for this study captures whether a student reported that during the school year (coded as *yes* = 1, *no* = 0) anyone had called them an insulting or bad name based on their race, religion, ethnic background or national origin, disability, gender or sexual orientation. Although this measure is also disaggregated by the type of hate speech, this study uses the aggregated version due to sample size limitations.

#### Authoritative school climate

This study operationalized two distinct facets of authoritative school climate consistent with prior research (Gee et al., 2022). The first is a school disciplinary structure index constructed from four items using factor analysis. These four items captured the extent to which adolescents agreed, on a 4-point scale from 1 (*strongly agree*) to 4 (*strongly disagree*), that (a) their school’s rules were fair, (b) the punishment for breaking rules was the same for everyone, (c) rules were strictly enforced, or (d) students knew the punishment if rules were broken. Ordinal reliability for the items was 0.87. Factor analysis based on a polychoric correlation matrix yielded one factor (eigenvalue = 2.75) with each item loading high on this one factor (loadings were >0.78). The resulting index constructed using factor analysis on the four underlying items was scaled with a mean of zero and standard deviation of one. Higher scores on the index indicate that students perceive their schools as having stronger levels of strict, yet fair disciplinary rules.

The second is a student support index constructed from four items using factor analysis. Three of the items captured the extent to which adolescents agreed, on a 4-point scale from 1 (*strongly agree*) to 4 (*strongly disagree*), that there was a teacher or adult at school who (a) really cared about them, (b) listened to them when they had something to say, or (c) told them when they did a good job. The other item asked adolescents to report on the same 4-point scale the extent to which teachers at their school treated students with respect. Ordinal reliability for the items was 0.91. Factor analysis based on a polychoric correlation matrix yielded one factor (eigenvalue = 2.80) with each item loading high on this one factor (loadings were >0.94). The resulting index constructed using factor analysis on the four underlying items was scaled with a mean of zero and standard deviation of one. Higher scores on the index indicate that students perceive their schools as having stronger support from adults.

#### Caring peers and guardianship

Consistent with prior research that examines Opportunity Theory and Asian American victimization (Gee & Cooc, 2019), this study includes the presence of caring peers and guardianship in the form of student reported measures of school security. Similar to the student support index capturing the presence of supportive adults, a caring peer index was constructed based on factor analysis of three items: students responded whether they agreed on 4-point scale, ranging from 1 (*strongly agree*) to 4 (*strongly disagree*), whether they had a friend at school to talk to, cared about their feelings, or cared for what happened to them. As with the other indices, this index was constructed using factor analysis and was scaled with a mean of zero and standard deviation of one.

#### Exposure to victimization

Several victimization exposure-related conditions were also included according to the literature (Popp, 2012), including the number of extracurricular activities that students participated in (a summed score ranging from 0 to 3, based whether a student participated in four activities: athletics, arts, academic clubs, and school government) as well as the presence of guns, fights and gangs at school (measured dichotomously: 1 = *yes*, 0 = *no*).

#### Controls

Consistent with prior studies using the SCS data (Gee et al., 2022), controls were included for students’ demographic background characteristics, including: (a) gender; (b) parental education level (whether the parent had a college education or not); (c) academic grades (a categorical variable documenting whether a student had mostly A’s, B’s or C’s or below); (d) whether the student attended a public versus private school; and (e) whether the student was in middle school or high school. Finally, indicators were included for survey year to account for temporal differences in hate speech as well as indicators for region (Northeast, Midwest, South and West) to account for regional differences in hate speech. The controls help reduce bias and confounders in estimating the relation between hate speech and authoritative school climate.

#### Analytic strategy

Logistic regression was used to model the relationship between selected school contexts and the probability of experiencing hate speech. More formally, the following model for individual *i* was fit to data:

$${logit}\left({p}_{i}\right)=\alpha +\beta {X}_{i}+{Z}_{i}$$

where ${logit}\left({p}_{i}\right)$ is the log odds (i.e., $\mathrm{ln}(\frac{{p}_{i}}{1-{p}_{i}})$) of hate speech. ${X}_{i}$ represents a vector of measures capturing authoritative school climate, guardianship and exposure predictors whose relationship to hate speech is captured in coefficient vector *β*. Finally, ${Z}_{i}$ represents a vector of controls. For ease of interpretation, coefficient estimates (i.e., ${e}^{\beta })$ are exponentiated and presented as odds ratios.

Given the complex sampling design of the SCS, survey weights and design information (strata and primary sampling unit) were incorporated. Standard errors were estimated using Taylor linearization, the prescribed method for analyses using the SCS. This study adopted a significance level of α = 0.05 with which to test the null hypothesis that selected predictors were unrelated to the outcome. To handle missing data (present in 15 variables, ranging from 0% to 23%), this study used multiple imputation by chained equations (MICE) where 10 datasets were imputed. Models were fit to data within each imputed dataset and the results were pooled together using Rubin’s rules.

## Results

### Descriptive Statistics

Table 1 displays descriptive statistics for the sample pooled across all three waves of data (Supplemental Table A1 provides descriptive statistics disaggregated by wave). Based on the pooled descriptive statistics, 7 percent of Asian American students reported that they were victims of hate speech in the past school year. Students in the sample attended schools with higher than average disciplinary structure (*M* = 0.22; *SD* = 0.86) and student support (*M* = 0.13; *SD* = 0.46). Students also attended schools with average peer support (*M* = 0.02; *SD* = 0.82) and reported, on average, about 5 security features present in their schools. Regarding exposure-related measures, roughly 1% of Asian American students in the sample reported the presence of guns in their schools as well as involvement in physical fights while a larger proportion (4%) reported gangs at school. In terms of background demographic characteristics, the sample was on average, 14.8 years old and 52% were male. The majority of the sample (71%) had college-educated parents and attended a public school (90%).

**Table 1:** Weighted Descriptive Statistics on a Sample of Asian American Adolescents from the School Crime Supplement (SCS) to the National Crime Victimization Survey (NCVS) (2015, 2017, and 2019) (*n* = 938)

	Mean or proportion^a^	Standard deviation
Victim of hate speech (%)	0.07	0.24
Authoritative school climate		
Disciplinary structure index	0.22	0.86
Student support index	0.13	0.46
Caring peer index	0.02	0.82
Guardianship		
Number of school security features	5.69	1.53
Exposure		
Students brought guns to school (%)	0.01	0.11
Involved in physical fights at school (%)	0.01	0.11
Gangs at school (%)	0.04	0.18
Number of extracurricular activities	1.05	0.95
Skipped class (past 4 weeks) (%)	0.04	0.19
Age (in years)	14.80	1.74
Male (%)	0.52	0.48
Parents have college education or above (%)	0.71	0.43
Attends a public school (%)	0.90	0.29
In middle school (grades 6–8) (%)	0.39	0.47
Mostly A’s (%)	0.69	0.44
Mostly B’s (%)	0.27	0.43
Mostly C’s or below / Other (%)	0.04	0.18
Northeast (%)	0.23	0.40
Midwest (%)	0.15	0.34
South (%)	0.26	0.42
West (%)	0.37	0.46

Descriptives based on non-imputed data. Listwise deletion was used to handle missing data.

^a^For dichotomous variables (1 = yes; 0 = no) the decimal form (e.g., 0.07) of the percent (7%) is presented in the table. This represents the proportion of the sample with a specific characteristic or experience.

### Hate Speech Prevalence

**Fig. 1:**
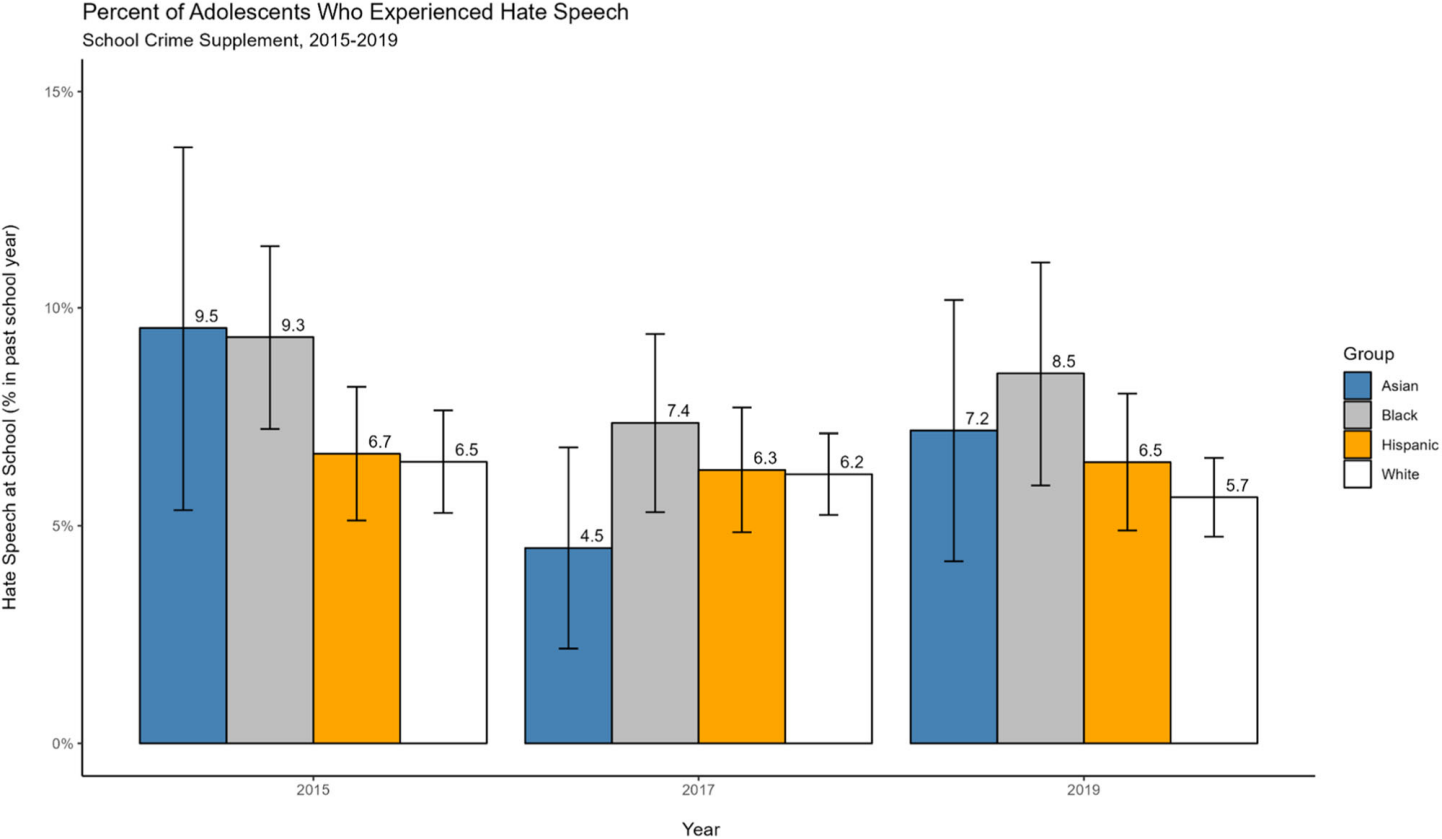
Percent of Adolescents Who Experienced Hate Speech by Race and Ethnicity. Estimates based on survey weighted data pooled across 10 imputed datasets. Error bars represent 95% confidence intervals (CIs)

Figure 1 displays the prevalence of hate speech disaggregated by race and ethnicity in 2015, 2017, and 2019. For Asian American youth, rates were approximately 9.5%, 4.5% and 7.2% in each wave respectively, averaging about 7% across all waves. This suggests that, on average, about 1 out of every 15 Asian American students reported being a victim of hate speech. The change was stable from wave to wave based on non-significant coefficient estimates on a set of year indicators from regression models predicting the relationship between survey year and hate speech (β = 0.05; *p* = 0.06 for the difference between 2015 versus 2017; β = −0.02; *p* = 0.39 for the difference between 2017 versus 2019; β = 0.03; *p* = 0.25 for the difference between 2015 versus 2019). While the percentage of Asian American youth who reported hate speech was slightly higher in 2015 versus the percentage from other racial and ethnic backgrounds (0.2, 2.8, and 3.0 percentage points higher versus Black, Hispanic or White students, respectively), these differences were not statistically significant. In 2017, rates for Asian American students were the lowest relative to all other racial and ethnic groups and in 2019, rates were lower relative to Black students, but higher than for Hispanic or White students. However, for both 2017 and 2019, rates did not statistically differ between Asian American students and the other racial and ethnic groups.

**Fig. 2:**
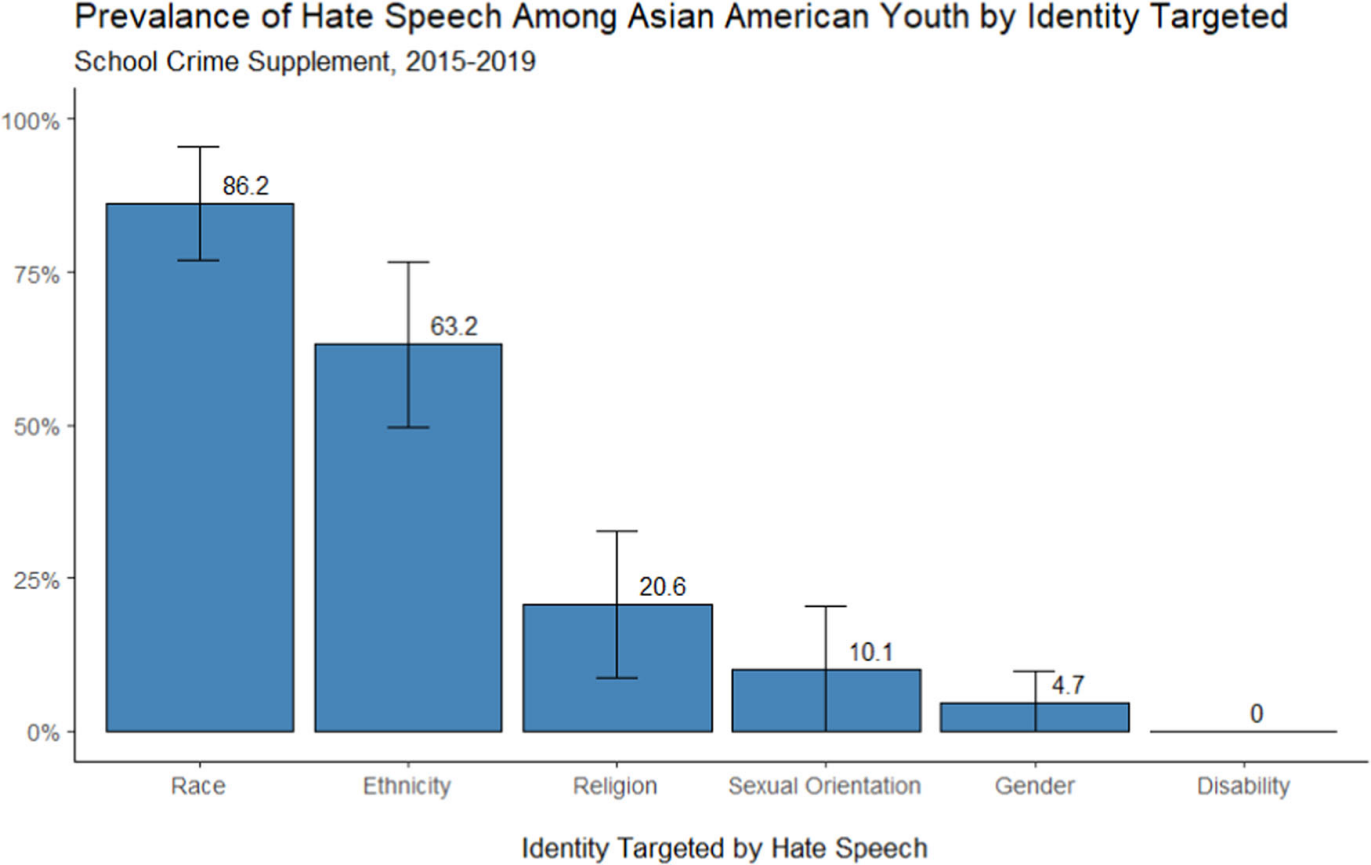
Prevalence of Hate Speech Among Asian American Youth by Identity Targeted. *Note*. Estimates based on survey weighted data pooled across 10 imputed datasets. Error bars represent 95% confidence intervals (CIs)

Figure 2 focuses on the subsample of Asian American youth who experienced hate speech and displays how frequently their different identities (e.g., racial, ethnic, religious, etc.) were targeted by such speech. Due to the small sample sizes in each wave, proportions for the entire sample of Asian American youth pooled across survey years are reported. As shown, the majority of Asian American youth who experienced hate speech reported that they were targeted because of their race (86%) followed by their ethnicity (63%). Relatively fewer youth who experienced hate speech, about 1 in 5, were targeted due to their religion (21%). Both sexual orientation (10%) and gender (4.7%) were the least common identities targeted by hate speech and no Asian American youth reported experiencing hate speech based on disability status.

### Effects of Disciplinary Structure, Support, Guardianship, and Exposure

Table 2 presents results establishing whether the measures of disciplinary structure, support, guardianship, and exposure relate to hate speech, first without controls (Model 1) and then with relevant controls (Model 2). As shown, prior to controlling for demographics, Asian American students reporting that they attend schools with stronger disciplinary structure and student support had lower odds of experiencing hate speech (*OR* = 0.69, 95% CI [0.47, 0.99], *p* < 0.05; and *OR* = 0.66, 95% CI [0.45, 0.97], *p* < 0.05, respectively). Both of these relationships—both in terms of magnitude and statistical significance—remain robust to the inclusion of individual controls. While students with higher levels of caring peers also experienced lower odds of hate speech (*OR* = 0.82, 95% CI [0.65, 1.02], *p* > 0.05), zero effects could not be ruled out net of controls. Similarly, the estimate on the guardianship variable, in the form of school security measures, indicated higher odds of hate speech, but zero effects could not be ruled out. Beyond a school’s authoritative climate, students who were involved in fights were also more likely to experience hate speech (*OR* = 4.66, 95% CI [1.10, 19.76], *p* < 0.05), a result which remains consistent between models.

**Table 2:** Logistic Regression Models Predicting the Odds of Experiencing Hate Speech Among Asian American Adolescents

	Model 1		Model 2	
	*OR*	*SE*	*OR*	*SE*
Authoritative school climate				
Disciplinary structure index	0.69^*^	(0.13)	0.69^*^	(0.13)
Student support index	0.66^*^	(0.13)	0.65^*^	(0.13)
Caring peer index	0.78^*^	(0.09)	0.82^~^	(0.09)
Guardianship				
Number of school security features	1.19	(0.11)	1.18	(0.13)
Exposure				
Students brought guns to school	2.00	(1.47)	2.17	(1.72)
Involved in physical fights at school	4.42^*^	(3.07)	4.66^*^	(3.41)
Gangs at school	3.87^*^	(2.37)	3.30^~^	(2.06)
Number of extracurricular activities	1.06	(0.21)	1.13	(0.23)
Skipped class (past 4 weeks)	1.14	(0.77)	0.83	(0.74)
Controls				
Age (in years)			0.82	(0.12)
Male			1.34	(0.43)
Parent education level (some college or above)			1.28	(0.47)
Attends a public school			6.92	(8.44)
In middle school (grades 6–8)			0.68	(0.34)
Grades (ref: A’s)				
B’s			1.09	(0.40)
C’s or below / Other			4.32^*^	(2.56)
Region & Year Fixed Effects	Yes		Yes	
Observations (unweighted)	938		938	

OR Odds ratio. Models based on ten imputed data sets, where missing data were estimated using chained equations. Models also incorporate survey weights and include fixed effects for survey year and region. Standard errors (SE) were estimated directly using Taylor series linearization.

*p < 0.05; ^~^*p* < 0.10.

To further compare the relative importance of each of the significant predictors in Model 2, the untransformed coefficient estimates (expressed in their original logit metric) were multiplied by their respective standard deviations to determine the association between a 1 SD change in the predictors and a change in the logged odds of the outcome (Pampel, 2000). These results show that a 1 SD increase in school disciplinary structure and student support have comparable associations with a decline in the occurrence of hate speech—both are associated with a lower logged odds of hate speech by approximately 0.35. On the other hand, involvement in physical fights has an opposite effect which is slightly lower in magnitude. A 1 SD increase in involvement in physical fights is related to an increase in the logged odds of hate speech by roughly 0.27.

**Fig. 3:**
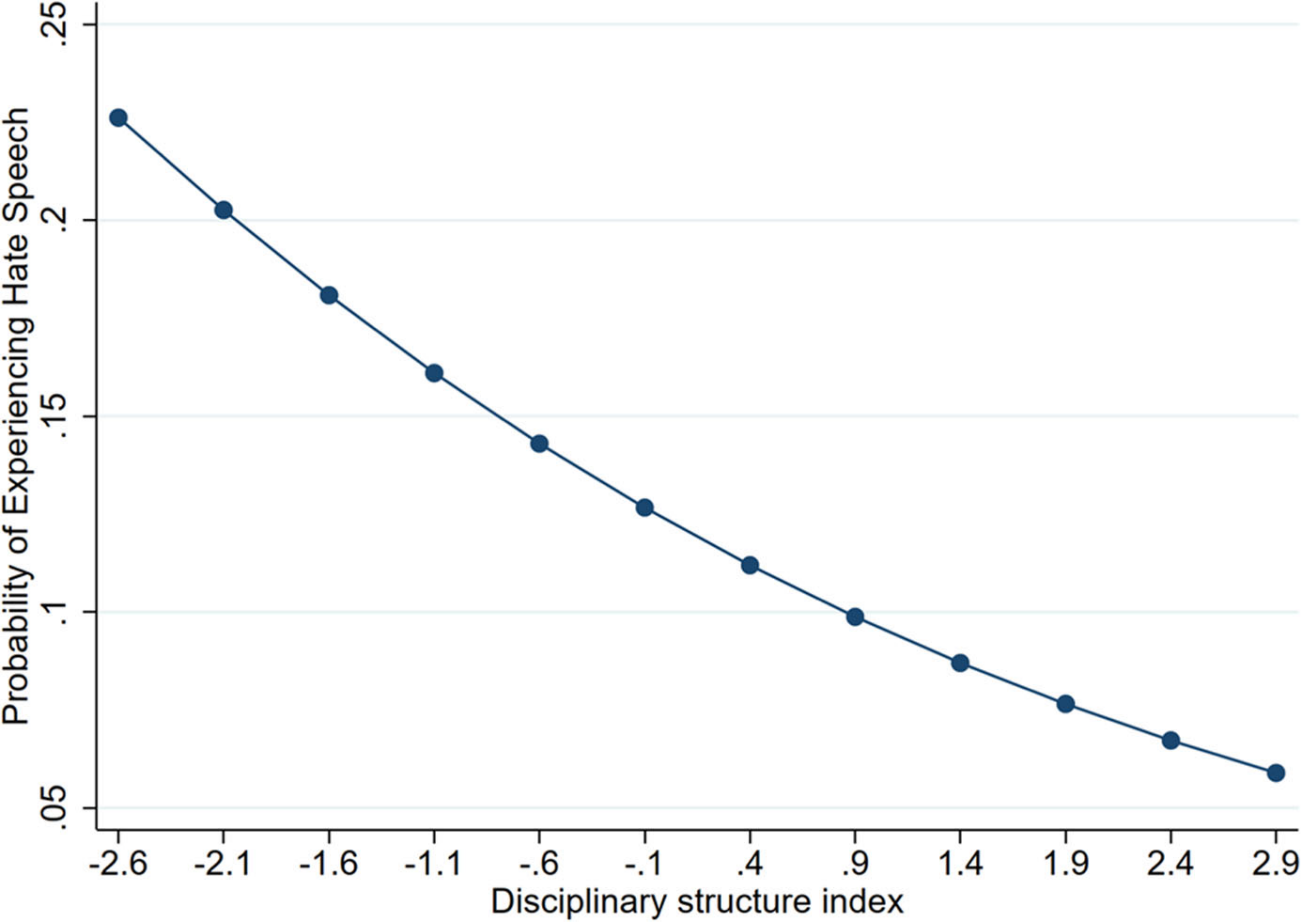
Association Between Disciplinary Structure and the Probability of Experiencing Hate Speech. The disciplinary structure index captures the extent to which students feel that their school’s rules are strict, yet fair (e.g., school rules are strictly enforced and the punishment for breaking such rules are the same for everyone) with increasing index values indicating higher levels of disciplinary structure. The values of the index are bounded by the maximum and minimum observed in the sample

**Fig. 4:**
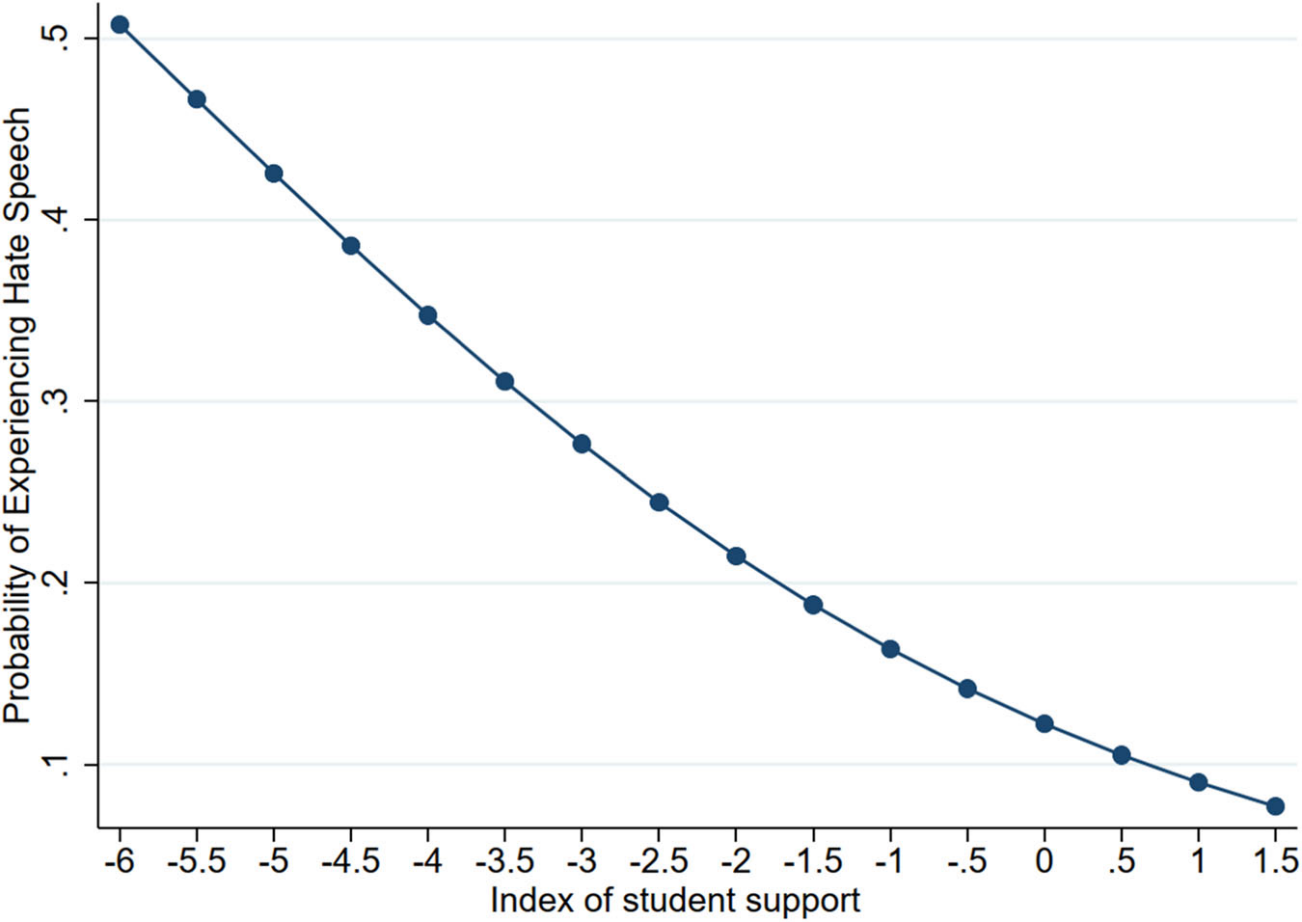
Association Between Student Support and the Probability of Experiencing Hate Speech. The index of student support captures the extent to which students feel supported by an adult at school (e.g., an adult cares about them) with increasing index values indicating higher levels of support. The values of the index are bounded by the maximum and minimum observed in the sample

Results from Model 2 are visually displayed in terms of fitted probabilities that depict how disciplinary structure and student support relate to the estimated probability of experiencing hate speech (Figs. 3 and 4). As shown in Fig. 3, increases in disciplinary structure are related to decreases in the probability of experiencing hate speech. This model predicts that, on average, students reporting the lowest levels of disciplinary structure (i.e., −2.6 on the disciplinary structure index scale) will experience about a 1 in 5 chance (22%) of experiencing hate speech. In contrast, those in schools with the strongest levels of disciplinary structure (i.e., 2.9 on the disciplinary structure index scale), face about a 1 in 20 chance (5.8%). Also, students who report the lowest levels of student support at school have nearly a 50% probability of experiencing hate speech, while their counterparts in schools with the highest levels experience roughly an 8% probability (Fig. 4). Thus, the probability of experiencing hate speech is nearly 6.25 times higher for students with the lowest levels of support relative to their peers with the highest level of support. Finally, for students who engaged in fights, their predicted probability of encountering hate speech is roughly 30%, compared to 12% for their counterparts who did not engage in fights.

## Discussion

Although hate against Asian American communities is longstanding and systemic, a strong confluence of recent events—the pandemic, coupled with social and political rifts—has intensified this hate. Schools have not been immune to the influence of these broader societal strains and even prior to the pandemic, race-related discrimination in schools was more prevalent among Asian American high school students relative to all other racial groups (Cooc & Gee, 2014). However, not only is it rare for studies to examine how school contexts are associated with hate speech incidents, but there are no studies, to the authors’ knowledge, that explicitly focus on how Asian American youth encounter such hate in schools at the national level, especially prior to the pandemic. This study helps overcome this oversight and has particular urgency and relevance given the recent swell of anti-AAPI hate.

This study’s findings establish that, on average, about 1 in 15 Asian American adolescents were targets of hate speech between 2015 and 2019, with rates remaining stable across the same time period. Further, there were no significant differences in rates between Asian American adolescents and those from other races and ethnicities. Given this baseline, it is important for future studies that will use the SCS 2022 and beyond to determine whether these trends significantly changed. This study also found that stronger authoritative climates were associated with reductions in the incidence of hate speech. In particular, higher levels of disciplinary structure in the form of school rules alongside the presence of supportive adults at school are linked to lower probability of Asian American adolescents experiencing hate speech at school. On the other hand, consistent with opportunity theory, students who engaged in school fights faced an increased probability. These results are consistent with authoritative disciplinary and opportunity theories, demonstrating how broadly applicable these theories can be to the hate speech experiences of Asian American youth. Further, these findings build upon and complement the extant empirical evidence on the victimization experiences of Asian American youth (Gee & Cooc, 2019; Hong et al., 2014; Peguero & Williams, 2013). In particular, this study’s finding that physical fights were positively associated with hate speech are consistent with findings from Gee and Cooc (2019) who found that students who engaged in physical fights were more likely to experience social victimization. However, in contrast with the findings of Gee and Cooc (2019), who found that neither support nor disciplinary structure were related to physical or social victimization, this study shows that both aspects of authoritative climate were linked to reductions in hate speech. This finding is notable as it suggests that effectiveness of solutions aimed at mitigating school violence built around strengthening an authoritative school climate may not be wholly effective in stemming all forms of school violence, but may depend on the type of violence that students encounter. Future research can examine why different underlying dimensions of authoritative school climates function more or less effectively to influence a range of victimization experiences, including bullying alongside hate speech.

Importantly, these findings offer critical insights for interventions aimed at hate-speech reduction. Recent hate-speech interventions targeted to adolescents have focused on intervening to promote counter speech, a way of discouraging hate speech by pointing out logical flaws or using facts to counter misinformation (Wachs et al., 2023). HateLess is an intervention that was developed to increase counter speech, as well as increase empathy towards victims of hate speech and self-efficacy in intervening when hate speech is occurring (Wachs et al., 2023). HateLess is composed of five modules delivered over a one week timeframe and fosters an in-depth understanding of what hate speech is and how to effectively counter it. Overall, HateLess has direct small to moderate effects on counter speech, empathy, and self-efficacy; however, the long-term effects (greater than one month) are unknown (Wachs et al., 2023). Given that the success of interventions like HateLess depend on the school social context as well as available resources, it will be important for schools to plan ways to overcome barriers that could hinder the successful implementation of such programs. For example, schools that have limited resources can leverage partnerships with local youth or community-based organizations to collaboratively deliver and implement interventions like HateLess. More challenging, however, are the political and social tensions that have spilled over into schools, placing them front and center over debates over what the kinds of social issues that can and cannot be discussed or incorporated into school programming or curricula—as a result, addressing hate speech that involves race and systemic racism could be more challenging in specific communities (Walker, 2023).

Beyond interventions like HateLess, positive classroom climate and social skills—specifically, perspective-taking, prosocial behavior, and assertiveness—could positively relate to counter speech (Wachs et al., 2023). Empirically, findings on a sample of 3225 youth (grades 7–9) in German and Swiss schools demonstrated that classroom climate and social skills had small to moderate effects on counter speech (Wachs et al., 2023). Further, stronger classroom climates (measured by the quality of relationships with classmates) were associated with higher levels of social skills, suggesting that improving classroom climate and student social skills could protect adolescents from hate speech.

Taken together, this study’s findings, alongside evidence to promote counter speech as well as the role of classroom climate in protecting students from hate speech, suggests that a multi-tiered socioecological approach—one that interweaves interventions at the individual, classroom and school-levels—will be important in reducing hate speech. In practice, one model that has been used to address bullying, which could also be applied specifically to hate speech, is a two-tiered approach. At its foundation, the model provides universal supports (e.g., classroom lessons, staff-led bystander intervention training, etc.) to all individuals involved—victims, perpetrators, and bystanders—while at the same time, directing more intensive interventions to address the behaviors of the perpetrators and behavioral supports, like counseling, for victims (Nickerson, 2019). The evidence generated from this study also points to critical schooling conditions that may enhance, or moderate, the efficacy of individual and classroom based interventions. For instance, the success of counter speech interventions may depend on the strength of the authoritative climate and the presence of caring adults in the school; conversely, those efforts may be more challenging if students face physical fights. Developing and testing how multi-tiered approaches can address hate speech, especially culturally relevant strategies specific to the experiences of Asian American youth, are areas for further research.

In terms of implications for policy, while schools often have policies addressing bullying, harassment and discrimination that include definitions of hate speech, this study’s findings underscore the need to strengthen guidance that undergirds such policies. Guidance can include the promotion of anti-bullying prevention and training activities, the explicit acknowledgment of hate against Asian American youth as well as more detailed data collection and reporting to identify patterns in such incidents. For instance, California’s Assembly Bill (AB) 2291: Bullying Prevention, enacted in 2019, amended the existing Education Code and requires school districts to adopt “procedures for preventing acts of bullying, including cyberbullying” and to publicly post online training materials to support educators and student support staff in preventing bullying (California Department of Education, 2022). However, these materials lack specific acknowledgement of bullying or hate speech against Asian American youth, nor guidance about specific data collection on and reporting of these incidents. In contrast to California, Iowa’s law on bullying and harassment (Section 280.28) specifies that schools report bullying and harassment data which is a step in the right direction; however, such incidents do not note the race of the victims which could limit the usefulness of such data in addressing and preventing victimization of and hate against Asian Americans. Finally, merely incorporating guidance and data reporting to strengthen existing policies may be insufficient to actually prevent incidents of hate speech unless schools also develop clear systems and procedures to ensure the underlying policies will be implemented on the ground with strong fidelity (Hall, 2017).

Limitations of this study include the inability to disaggregate the SCS data into Asian subgroups to understand their unique experiences of hate speech—this is a common limitation inherent in all studies of Asian American adolescents that leverage large-scale nationwide data and will require broader change at the federal level to incorporate more fine-grained sampling techniques to capture representative subpopulations. Second, this study can only address how authoritative climates are correlated with hate speech and is thus limited in establishing a causal link. This limitation can be overcome in future work by leveraging experimental or quasi-experimental designs. Third, due to sample size limitations, this study is unable to tease out trends in hate speech against different targeted identities; however, given that the majority of the sample experienced hate speech due to their race (86%) and ethnicity (63%), this study’s findings are, in large part, picking up trends in hate speech against Asian American youth’s racial and/or ethnic identities. Fourth, the measures used are subject to self-report bias and future studies would benefit by including data collected from administrative records and/or peer assessment, where feasible. Finally, quantitative data can only capture one piece of a complex story around hate speech. Future studies could benefit from complementary qualitative and mixed-methods designs that capture the lived experiences of youth experiencing hate in schools. Qualitative insights that center Asian American youth’s voices—especially with respect to the kinds of supports and strategies that they leverage to cope with bullying—can be instrumental in the design and delivery of culturally relevant anti-bullying and counter speech interventions, leading to robust interventions that are more relevant and effective.

## Conclusion

Although hate directed at Asian Americans has existed for generations, a prominent gap in the extant literature base is how pervasive hate speech was *prior* to the pandemic among Asian American adolescents and ways in which schooling contexts could perpetuate it or offer protection from it. This study is significant because it reveals that Asian American youth were victims of hate speech at statistically similar rates as their peers before the pandemic. This study further identifies schooling factors that strongly relate to whether Asian American youth are victims of hate speech; not only are the authoritative climates of schools related to a reduction in being victimized by hate speech, but engaging in school fights relates to a significant increase in experiencing hate speech. These conditions—especially authoritative school climates—are malleable features that can be shaped directly by larger school-climate related policies, programs and interventions. Efforts to alter schooling conditions to promote stronger authoritative climates and reduce exposure risks, like school fights, hold considerable promise in protecting Asian American youth from hate speech at school.

### Supplementary Information

Supplemental Table A1
