# Supplementary material for: Hate Speech Against Asian American Youth: Pre-Pandemic Trends and The Role of School Factors
Source: J Youth Adolesc. 2024 May 4;53(9):1941–52. doi: 10.1007/s10964-024-01987-8 (PMC11333554; doi:10.1007/s10964-024-01987-8)
Supplement: Supplementary file 1 — Supplemental Table A1 [file 10964_2024_1987_MOESM1_ESM.rtf]

Supplemental Table A1
Weighted Descriptive Statistics on a Sample of Asian American Adolescents from the School Crime Supplement (SCS) to the National Crime Victimization Survey (NCVS) (2015, 2017, and 2019) Disaggregated by Wave

	2015	2017	2019	
	Mean or proportiona	SD	Mean or proportiona	SD	Mean or proportiona	SD	
Victim of hate-related words (%)	0.12	0.31	0.04	0.20	0.05	0.19	
Disciplinary structure index	0.19	0.82	0.23	0.91	0.24	0.85	
Student support index	0.19	0.29	0.04	0.65	0.17	0.29	
Caring peer index	0.06	0.57	-0.00	0.89	-0.00	0.92	
Number of school security features	5.72	1.43	5.59	1.47	5.80	1.69	
Guns at school (%)	0.01	0.07	0.02	0.12	0.02	0.12	
Involved in physical fights at school (%)	0.01	0.11	0.00	0.06	0.03	0.15	
Gangs at school (%)	0.06	0.22	0.01	0.11	0.05	0.21	
Number of extracurricular activities	1.00	0.99	0.99	0.91	1.17	0.91	
Skipped class (past 4 weeks)	0.04	0.18	0.04	0.18	0.06	0.21	
Age (in years)	14.71	1.74	14.86	1.77	14.84	1.67	
Male (%)	0.51	0.49	0.53	0.48	0.53	0.46	
Parents have college education or above (%)	0.64	0.47	0.74	0.43	0.77	0.39	
Attends a public school (%)	0.91	0.28	0.90	0.28	0.88	0.30	
In middle school (grades 6-8) (%)	0.41	0.48	0.39	0.47	0.37	0.44	
Mostly A's (%)	0.70	0.45	0.70	0.44	0.67	0.43	
Mostly B's (%)	0.23	0.41	0.28	0.43	0.30	0.42	
Mostly C's or below / Other (%)	0.07	0.24	0.02	0.14	0.03	0.15	
Northeast (%)	0.21	0.40	0.18	0.37	0.31	0.43	
Midwest (%)	0.14	0.34	0.12	0.31	0.19	0.36	
South (%)	0.26	0.43	0.28	0.44	0.22	0.38	
West (%)	0.39	0.48	0.42	0.48	0.27	0.41	
Note. Descriptives based on non-imputed data and listwise deletion was used to handle missing data.

a For dichotomous variables (1 = yes; 0 = no) the decimal form (e.g., 0.12) of the percent (12%) is presented in the table. This represents the proportion of the sample with a specific characteristic or experience.
